# Facing problems in radiotherapy for breast cancer patients in Yogyakarta, Indonesia: A cohort retrospective study

**DOI:** 10.1002/cam4.5634

**Published:** 2023-01-20

**Authors:** Fithria Dyah Ayu Suryanegara, M. Rifqi Rokhman, Ericko Ekaputra, Lisa Aniek de Jong, Didik Setiawan, Geertruida H. de Bock, Maarten Jacobus Postma

**Affiliations:** ^1^ Department of Health Sciences, University Medical Center Groningen University of Groningen Groningen The Netherlands; ^2^ Department of Pharmacy Universitas Islam Indonesia Yogyakarta Indonesia; ^3^ Faculty of Pharmacy Universitas Gadjah Mada Yogyakarta Indonesia; ^4^ Dr. Sardjito General Hospital Yogyakarta Indonesia; ^5^ Department of Radiology, Faculty of Medicine, Public Health and Nursing Universitas Gadjah Mada Yogyakarta Indonesia; ^6^ Faculty of Pharmacy Universitas Muhammadiyah Purwokerto Purwokerto Indonesia; ^7^ Department of Epidemiology, University Medical Center Groningen University of Groningen Groningen The Netherlands; ^8^ Department of Economics, Econometrics & Finance, University Medical Center Groningen University of Groningen Groningen The Netherlands; ^9^ Department of Pharmacology and Therapy, Faculty of Medicine Universitas Airlangga Surabaya Indonesia; ^10^ Centre of Excellence in Higher Education for Pharmaceutical Care Innovation Universitas Padjadjaran Bandung Indonesia

**Keywords:** breast cancer, dose fractionation, patient compliance, radiation oncology, radiotherapy

## Abstract

**Background:**

The aim of this study is to explore problems in radiotherapy for breast cancer patients in Yogyakarta, Indonesia, focusing on overall treatment time (OTT) and completion rate.

**Methods:**

A retrospective cohort study was conducted based on data from the Insurance Unit at a tertiary hospital in Yogyakarta, Indonesia. The study included all female outpatients with breast cancer who were treated with radiotherapy from January to December 2017 and met the inclusion criteria. The primary outcomes were OTT and completion rate. The secondary outcomes included the number of radiotherapy fractions, radiotherapy doses, number of radiotherapy interruption days, and reasons for radiotherapy interruption. The chi‐squared and Mann–Whitney *U* tests were used to assess the differences in outcomes between two insurance schemes (JKN‐PBI (Beneficiaries of Health Insurance Contribution Assistance) and JKN‐NON‐PBI (Non‐Beneficiaries of Health Insurance Contribution Assistance)).

**Results:**

The sample included 285 breast cancer patients (mean age: 53 years). The median OTT was 38 days (IQR: 17–48 days), with 123 (43.2%) patients having prolonged OTT. The completion rate was 57.9%. No significant differences in OTT (44.4% vs. 35.7%, *p* = 0.445) and completion rate (57.2% vs. 61.9%, *p* = 0.569) were found between the JKN‐NON‐PBI and JKN‐PBI groups, respectively. In all, the data reported 3,022 interrupted days of radiotherapy across a total of 227 patients. The most common reason for radiotherapy interruption was unknown.

**Conclusion:**

There are problems in timely delivery and low completion rate of radiotherapy among breast cancer patients in Indonesia. There are no significant differences in OTT and completion rate between the insurance schemes.

## Graphical abstract

There are problems in timely delivery and low completion rate of radiotherapy among breast cancer patients in Indonesia. There are no significant differences in overall treatment time and completion rate between the insurance schemes.

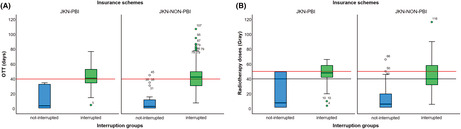

## 1 INTRODUCTION

In Indonesia, which is classified among the low‐ and middle‐income countries (LMIC), breast cancer is the most common cancer, with a prevalence of 201,143 and a mortality rate of 9.6% in 2020.^1^ Breast cancer patients require personalized multimodality treatment.^2^, ^3^ However, effective cancer management can be challenging in LMIC, due to limitations in finances, resources, trained human resources, and infrastructure. Radiotherapy is used to reduce the risk of recurrence and mortality rate after breast‐conserving surgery and adjuvant endocrine therapy.^4^ In Indonesia, radiotherapy is performed for 53.7% of all breast cancer patients.^3^

Good clinical practice for radiotherapy dictates that complete treatment doses should be administered without interruption or prolongation of overall treatment time (OTT). Incompletion and prolongation OTT of radiotherapy results in negative clinical outcomes including increases the risk of local recurrence and inferior overall survival.^5^ In clinical practice, however, interruptions are unavoidable.^6^, ^7^ Even low levels of non‐compliance and prolongation of OTT result in negative clinical outcomes.^5^

Previous studies on nasopharyngeal cancer patients in Yogyakarta, Indonesia reported that the OTT was 15–17 days longer than the international standards.^8^ Few researchers have addressed OTT and radiotherapy completion rates among breast cancer patients.^9^, ^10^, ^11^, ^12^, ^13^, ^14^ In this study, we explore problems that breast cancer patients experience with radiotherapy, focusing on OTT and radiotherapy completion rates. We hypothesize that the average OTT of breast cancer patients in Indonesia is longer than standard, and the radiotherapy completion rate is suboptimal.

We also investigate the influence of insurance type on the OTT and completion rates. Two types of insurance schemes are applied in Indonesia since the enactment of Universal Health Coverage (UHC) in 2014: JKN‐PBI and JKN‐NON‐PBI. The JKN‐PBI scheme applies to people who are poor and/or disadvantaged. The insurance contributions of those covered by this scheme are paid by the government. Under the JKN‐NON‐PBI scheme, contributions are paid either by individuals themselves or by their employers.^15^

## 2 METHODS

### 2.1 Study design

This retrospective cohort study was conducted in a tertiary hospital in Yogyakarta, Indonesia. We analyzed claims data from the Hospital Insurance Unit of BPJS Kesehatan (Indonesia's National Health Insurance [NHI]) concerning consecutive series of breast cancer outpatients who underwent radiotherapy from January to December 2017. These data include information on patient demographics, insurance schemes, primary diagnoses, International Classification of Disease Ninth Revision Clinical Modification (ICD‐9‐CM) codes, Indonesian‐Case Based Groups codes, radiotherapy‐session attendance, and direct medical costs. This study was a part of another project evaluating the cost of treatment with radiotherapy for breast cancer in Yogyakarta, Indonesia. The study was reviewed and approved by the Medical Health Research and Ethics Committee (approval number KE/FK/0449/EC/2018), and it followed the Strengthening the Reporting of Observational Studies in Epidemiology (STROBE) checklist.^16^

### 2.2 Patients

The research population for this study included all breast cancer outpatients reported in the BPJS Kesehatan data claims, with the following eligibility criteria: female breast cancer with a primary intervention code of Z51.0 (radiotherapy), an INA‐CBG code of C‐3‐10‐0 (radiotherapy procedures), and undergoing treatment with the current procedural terminology for radiation treatment delivery (with the ICD‐9‐CM code of 92.24). In all, 334 patients were analyzed for inclusion. We followed the patients who had received radiotherapy in 2017 from the start date on which they received treatment until the last fractions received in 2017.^6^, ^17^ Patients who did not have full coverage under either the JKN‐PBI or the JKN‐NON‐PBI scheme in 2017 were excluded. To minimize selection bias, patients who received radiotherapy for only 1 month in January or December 2017 were excluded, as the fractions received in those months might have been part of a series of radiotherapy sessions from the preceding or subsequent year.

### 2.3 Radiotherapy treatment plan

The patients were treated with external beam radiotherapy in a 60‐Co or 6‐MV linear accelerator plane using two‐dimensional conventional radiotherapy, three‐dimensional conformal radiotherapy, or an intensity modulated radiation therapy technique. Patients were prescribed a dosing scheme consisting of 50 Gray (Gy), delivered in 25 fractions within a 5‐week period. Each radiotherapy fraction consisted of 2 Gy, with a booster doses of 10–20 Gy if needed.^17^

### 2.4 Study outcomes

The primary study outcomes were OTT and completion rate. The OTT was calculated from the time that radiotherapy was initiated to the final day of radiotherapy fractions for all patients with near/full completion of radiotherapy treatment and for those who had not completed treatment. Any radiotherapy fractions that were interrupted for more than 2 months were counted as a new radiotherapy treatment plan. The standard range of OTT for radiotherapy is between 35–40 days, with OTT exceeding 40 days regarded as prolonged.^6^, ^17^ The radiotherapy completion rate was classified into two categories of radiotherapy completion: (a) near/full completion was defined as patients who received at least 80% or more of the standard fractions of radiotherapy (≥20 fractions) and (b) non‐completion as patients who received less than 80% of standard fractions of radiotherapy (<20 fractions).^18^

The secondary outcomes were the number of radiotherapy fractions, radiotherapy doses, number of radiotherapy interruption days, and reasons for radiotherapy interruptions. The number of radiotherapy fractions administered to the breast cancer patients was estimated using the ICD‐9‐CM code 92.24. In the claims data of BPJS Kesehatan, one claim was filed for each radiation treatment. The radiotherapy fractions were calculated over the span of the OTT. Radiotherapy doses were estimated by multiplying the number of fractions by 2 Gy. The hospital's standard therapy guidelines for breast cancer patients prescribe radiotherapy to be delivered in doses of 2 Gy with total of 50 Gy.^17^, ^18^ A treatment interruption was defined as having missed at least one scheduled radiotherapy fraction.^19^ Saturdays and Sundays were not counted as treatment interruptions, as conventional radiotherapy fractionation schedules take weekends into account as a matter of course. These days were included for interruptions occurring either before or after the weekend. For example, if a patient experienced an interruption on Friday, the total interruption was counted as 3 days.^14^ The reasons for radiotherapy interruptions were divided into three categories: (a) public holidays, (b) machine breakdown, and (c) unknown. The category “unknown” includes all reasons other than public holidays and machine breakdown (e.g., patient non‐adherence, unplanned physician‐initiated cancelation, side effect of therapy experienced by patients, or financial issues).

### 2.5 Statistical analyses

Patient demographic characteristics were described as percentages for the two completion groups. OTT was reported as the median and interquartile range (IQR), along with the mean and standard deviation (SD). Radiotherapy completion rate was expressed as a percentage. Mann–Whitney *U* tests were used to evaluate differences in radiotherapy starting day between the two completion groups, as well as differences between the two insurance schemes in terms of OTT, number of fractions, and doses. The same tests were used to analyze OTT and radiotherapy doses in the two insurance schemes for patients who experienced radiotherapy interruptions. A Chi‐squared test was conducted to evaluate differences between the two completion groups in terms of age and insurance scheme, as well as differences between the two insurance schemes in terms of radiotherapy interruptions.

Multivariate logistic regression was used to assess the odds of OTT and the completion rate of radiotherapy. Factors included in the final model were age, insurance scheme, and starting day of radiotherapy. Statistical analysis was performed using IBM SPSS Statistics version 28 and Microsoft 365 Excel®. All tests were two‐sided, and results were considered statistically significant for *p*‐values < 0.05.

## 3 RESULTS

Between January and December 2017, radiotherapy was administered to 334 breast cancer outpatients. In all, 49 patients were excluded from the study: 43 patients who had radiotherapy sessions only in January or December 2017, four male patients, and two patients who had used both insurance schemes in the same year. The remaining 285 patients met the inclusion criteria (Figure S1). Patient characteristics for the overall population and by insurance scheme are presented in Table 1. Overall, the mean (SD) patient age was 53 years (10), and most of the patients started radiotherapy on Monday (31.2%). A total of 243 patients (85.3%) were covered by the JKN‐NON‐PBI scheme. The Chi‐squared and Mann–Whitney *U* tests indicated that there was no difference between the two insurance schemes in terms of age or starting day of radiotherapy (Table 1).

**TABLE 1:** Patient characteristics by insurance scheme

Characteristic	Overall (*n* = 285)	JKN‐NON‐PBI (*n* = 243)	JKN‐PBI (*n* = 42)	*p*‐value
Gender, *n* (%)				
Female	285 (100)	243 (100)	42 (100)	‐
Age (years), *n* (%)				
≤65	261 (91.6)	220 (90.5)	41 (97.6)	0.223
>65	24 (8.4)	23 (9.5)	1 (2.4)	
Mean (SD)	53 (10.0)	53.0 (10.0)	49.8 (8.0)	‐
Starting day of radiotherapy, *n* (%)				
Monday	89 (31.2)	77 (31.7)	12 (28.6)	0.539
Tuesday	79 (27.7)	70 (28.8)	9 (21.4)	
Wednesday	73 (25.6)	57 (23.4)	16 (38.1)	
Thursday	35 (12.3)	31 (12.8)	4 (9.5)	
Friday	9 (3.2)	8 (3.3)	1 (2.4)	

Abbreviation: SD, standard deviation.

### 3.1 OTT

**FIGURE 1:**
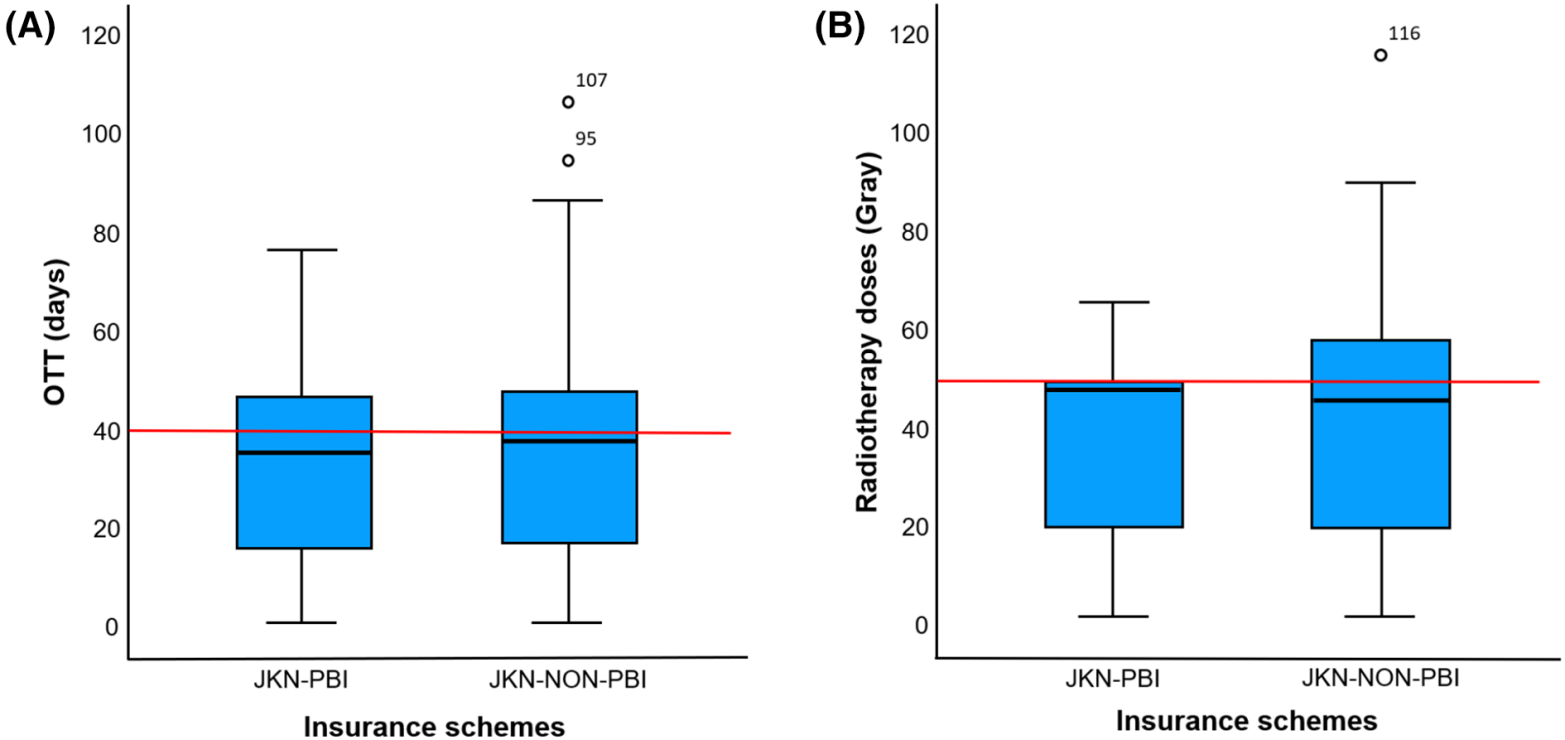
Distribution of overall treatment time (A) and radiotherapy doses (B) by insurance schemes. The dark lines in the boxes represent the median. The red line represents the maximum standard overall treatment time (40 days, A) and the standard number of radiotherapy doses (50 Gray, B). The lower and upper boundaries of the boxes represent the 25th and 75th quartiles, respectively, and the whiskers represent 1.5 times the interquartile range (IQR).

The median OTT for the overall study population was 38 days (IQR: 17–48 days). Prolonged OTT was nevertheless observed for 123 (43.2%) of the overall study population, 108 (44.4%) patients in the JKN‐NON‐PBI group and 15 (35.7%) patients in the JKN‐PBI group (Figure 1A). There was no significant difference of OTT between the two insurance schemes (*p* = 0.445; Table 2).

**TABLE 2:** Overall treatment time, number of fractions and radiotherapy doses

Outcomes	Overall (*n* = 285)	JKN‐NON‐PBI (*n* = 243)	JKN‐PBI (*n* = 42)	*p*‐value
OTT (days)				
Median (IQR)	38 (17–48)	38 (17–48)	36 (16–47)	
Mean (SD)	36 (21)	36 (21)	33 (21)	0.445
Completion rate (%)	165 (57.9)	139 (57.2)	26 (61.9)	0.569
Number of fractions				
Median (IQR)	24 (10–29)	23 (10–29)	24 (9–25)	
Mean (SD)	19 (11)	20 (11)	19 (11)	0.547
Doses (Gray)				
Median (IQR)	48 (20–58)	46 (20–58)	48 (18–50)	
Mean (SD)	39 (21)	39 (21)	37 (21)	0.547
Radiotherapy interruptions (%)	227 (79.6)	198 (81.5)	29 (69.0)	0.065

Abbreviations: IQR, interquartile range; OTT, overall treatment time; SD, standard deviation.

### 3.2 Completion rate

Patient characteristics by completion rate are presented in Table S1. Overall, 57.9% of patients completed 20 or more radiotherapy fractions. The mean age of these patients was quite similar across completion groups. According to the Chi‐squared and Mann–Whitney *U* tests, there were no statistically significant differences between the two groups in terms of patient age, insurance scheme, or starting day of radiotherapy (*p* = 0.633, *p* = 0.569, *p* = 0.493, respectively).

### 3.3 Number of fractions and doses of radiotherapy

The median number of radiotherapy fractions per patient was 24 fractions (IQR 10–29) and the median of radiotherapy doses was 48 Gy (IQR 17.5–50 Gy; Table 2). There are no significant differences of number of fractions and radiotherapy doses between the insurance groups (*p* = 0.547, *p* = 0.547, respectively). Overall, 120 patients received suboptimal radiotherapy doses: 42.8% patients in the JKN‐NON‐PBI group and 38.1% in the JKN‐PBI group (Figure 1B).

### 3.4 Interrupted days

In all, the data reported 3022 interrupted days of radiotherapy across a total of 227 patients (Table S2). The median number of interrupted days was 10 (IQR 6–17 days). Radiotherapy interruptions were more common in the JKN‐NON‐PBI group (81.5%) than the JKN‐PBI group (69.0%), with the difference approaching significance (*p* = 0.065; Table 2). The most common reason for radiotherapy interruption was “unknown,” accounting for 69% of all interrupted days, followed by machine breakdown (17.3%), and public holidays (13.7%).

**FIGURE 2:**
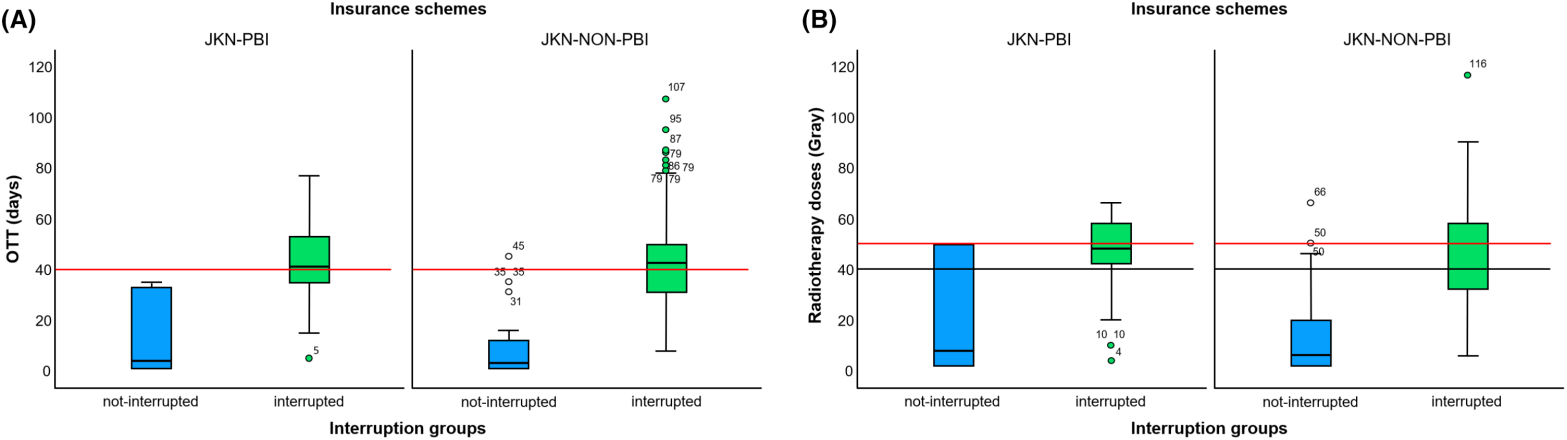
Distribution of overall treatment time (A) and radiotherapy doses (B) by insurance schemes, based on the occurrence of radiotherapy interruption. The dark lines in the boxes represent the median. The red line represents the standard overall treatment time (40 days, A) and the standard number of radiotherapy doses (50 Gray, B). The blue and green boxes represent the 25th and 75th quartiles, respectively, and the whiskers represent 1.5 times the interquartile range (IQR). Mann–Whitney U test result *p* = 0.001 for overall treatment time (A) and *p* = 0.004 for radiotherapy doses (B).

The OTT of patients with no radiotherapy interruptions was less than the standard for both insurance schemes, while many of those whose radiotherapy had been interrupted experienced prolonged OTT (Figure 2A). The OTT of the two insurance groups differed significantly, based on the occurrence of radiotherapy interruption (*p* < 0.05).

We also analyzed differences between these groups in terms of radiotherapy doses. Of the patients who experienced radiotherapy interruptions, 24.1% and 31.8% received suboptimal doses in the JKN‐PBI and JKN‐NON‐PBI groups, respectively. The total of radiotherapy doses that have been administered differed significantly between the two insurance schemes, based on the occurrence of radiotherapy interruption (*p* = 0.004; Figure 2B).

The potential factors associated with the OTT and radiotherapy completion rates are presented in Table 3. There were no significant differences in either OTT or completion rate according to age, insurance scheme, or starting day of radiotherapy.

**TABLE 3:** Multivariate logistic regression analysis of factors that may influence radiotherapy OTT and completion rate

Parameter	Variable	OTT		Completion rate	
		Odds ratio (95% CI)	*p*‐value	Odds ratio (95% CI)	*p*‐value
Age (years)	≤65	1 (ref)		1 (ref)	
	>65	0.833 (0.349–1.987)	0.680	0.852 (0.366–1.984)	0.710
Insurance scheme	JKN‐PBI	1 (ref)		1 (ref)	
	JKN‐NON‐PBI	1.351 (0.678–2.693)	0.393	0.788 (0.399–1.557)	0.493
Starting day of radiotherapy	Monday	1 (ref)		1 (ref)	
	Tuesday	1.181 (0.642–2.172)	0.593	1.092 (0.592–2.017)	0.778
	Wednesday	0.855 (0.385–1.897)	0.332	0.929 (0.497–1.738)	0.818
	Thursday	2.516 (0.590–10.734)	0.700	1.335 (0.597–2.986)	0.482
	Friday	1.351 (0.678–2.693)	0.213	0.852 (0.366–1.984)	0.710

Abbreviation: ref, reference.

## 4 DISCUSSION

To our knowledge, this is the first study to explore problems with radiotherapy experienced by breast cancer patients in Indonesia, focusing on OTT and radiotherapy completion rate. The median OTT was 38 days, which is within the standard range. However, around 40% of the patients had a prolonged OTT. The majority of patients (57.9%) had suboptimal radiotherapy completion rates. The high proportion of patients with prolonged OTT may be explained by the presence of radiotherapy interruptions in breast cancer patients. In total there were over 3000 interrupted days in 227 patients, of which around 70% with reason “unknown”. No significant differences were found for any of the measured outcomes between the insurance schemes.

Radiotherapy completion rates of breast cancer patients in Indonesia deviate from international standards. Our results contrast with findings reported for higher‐income countries, such as the United States and Spain, where radiotherapy completion rate among breast cancer patients were 97% and 99.3%, respectively.^9^, ^11^ This difference may be explained by the availability of abundant radiotherapy resources and the stricter medical supervision involved in the management of breast cancer treatment in developed countries.^9^, ^11^ For 261 million Indonesian people in 2017, 261 radiotherapy machines were needed to achieve a 1 MV machine per million population. While in that year there were only 66 units radiotherapy machines available, covering only 18.3% of the total need.^20^ Another reasonable explanation for the relatively high percentage of patients with a suboptimal completion rate is the use of radiotherapy for palliative care purposes or low adherence. However, this could not be confirmed since the exact indication for the radiotherapy could not be retrieved from the NHI data claim.

Prolonged duration radiotherapy treatment of more than 1‐week results in a 5% decrease of 5 years locoregional control rates.^14^ Our study showed a median duration of an interruption of 10 days. Patients who had experienced interruptions in radiotherapy were more likely to have significantly prolonged OTT compared with patients without interruptions (*p* < 0.05). Of the patients experiencing interruptions, 47.1% had low completion rates, as compared to 87.9% of those who did not experience any interruptions. Regardless of interruption, more than half (55.4%) of the breast cancer patients received suboptimal doses. It is important to note that no compensatory doses were administered for the interrupted days within this group. As reported in a previous study, interruption‐related compensatory doses of radiotherapy improve Tumor Control Probability by up to 11%.^21^

Interruptions of radiotherapy can be predictable and unpredictable. Although it is not possible to anticipate all potential interruptions when treatment is first prescribed. Of the interrupted days, 69% due to unknown reason. As reported in a previous study, the “no‐show” patients were categorized as non‐adherent patient.^19^ In the current study, however, we were unable to conclude that patient “no‐shows” were indeed due to non‐adherence because we did not interview the patients directly.

Socioeconomic status is known to be a substantial factor in the access to breast cancer treatment.^22^ Therefore, this current study investigated the influence of insurance schemes on OTT and completion rates for radiotherapy. There were no differences between the JKN‐PBI group and the JKN‐NON‐PBI group in terms of OTT, completion rate, or doses. Interruptions were more common in the JKN‐NON‐PBI group (approaching significance *p* = 0.065). This may be caused by financial difficulty of paying the insurance contributions in the JKN‐NON‐PBI group. It has been reported that 30%–40% of individuals covered by the JKN‐NON‐PBI scheme always delay on paying their monthly contributions.^23^ Those who do not pay are ineligible to receive any services in healthcare facilities, including radiotherapy.

Several procedures potentially minimize the effect of radiotherapy interruptions. In the event of treatment gaps due to equipment maintenance, or other service‐related issues, the patients concerned should be transferred to other equipment of the same type or to a corresponding item of equipment. In the case of unscheduled treatment interruptions or those resulting in planning deviations of just a few days, the patients concerned could be treated over the weekend. Alternatively, two treatments could be scheduled on the same day, with a minimum lag time of 6 h between fractions. If it is not possible to compensate for the interruptions with the accelerated treatment options mentioned above, it might be necessary to increase the total doses and/or the doses per fraction based on radiobiological calculation.^6^

Another solution is to deliver hypofractionation radiotherapy, which could consist of 16 or even five high‐dose fractions.^24^ The pitfall of the hypofractionation scheme is the way coverage is organized, which is based on the patients attendance. Therefore, the sum of total claim based on the number of radiotherapy fractions does not cover the total cost of the treatment. Moreover, it will make the planning more complex. Potential suggestions for improvement of radiotherapy in Indonesia include the BPJS Kesehatan to increase the claim (which has not been increased since 2014) and adopt single disease payment system not by fraction. Additionally, the Indonesian government needs to invest in radiotherapy by increasing the availability of radiotherapy machines and well‐trained health care professionals.

### 4.1 Limitations

Our study is subject to several limitations. The radiotherapy information was based on administrative NHI claims data, which might not capture all information on radiotherapy fractions and other clinical details. Moreover, the data could not be complemented with other information in case of missing data. For this reason, the number of fractions, radiotherapy completion rates, radiotherapy doses, and number of interruption days are subject to a relatively high probability of misclassification bias. Importantly, we were not able to collect information on the reasons for non‐completion and interruption of radiotherapy directly from the patients. Another potential source of bias is the cut‐off point that excluded breast cancer patients who had visited the radiotherapy department only in January or December, by underestimating OTT and completion rates. If we had excluded patients starting on November 21 or later (14 patients or 5% of the total study population), the result would have been less powerful. Another limitation of the study is that it was restricted to a single Class A General Hospital, which may not be representative of other types of hospitals in Indonesia. Further studies are needed to explore the issue of patient adherence with larger samples, longer follow‐up times, and different types of hospitals. However, our findings provide useful feedback that could help healthcare stakeholders develop a protocol for compensating for radiotherapy interruptions and educate patients about the radiotherapy treatment in order to improve their adherence.

## 5 CONCLUSION

This retrospective study explored problems relating to the radiotherapy treatment of breast cancer patients in Yogyakarta. Although the median OTT was within the standard range, around 40% of the patients had a prolonged OTT, and the completion rate was suboptimal. There were no significant differences between the two insurance schemes in terms of OTT, completion rate, and radiotherapy doses.

## AUTHOR CONTRIBUTIONS

**Fithria Dyah Ayu Suryanegara:** Conceptualization (equal); data curation (equal); formal analysis (equal); funding acquisition (equal); investigation (equal); methodology (equal); project administration (equal); resources (equal); validation (equal); writing – original draft (equal); writing – review and editing (equal). **M. Rifqi Rokhman:** Data curation (equal); formal analysis (equal); methodology (equal); writing – review and editing (equal). **Ericko Ekaputra:** Conceptualization (equal); data curation (equal); formal analysis (equal); methodology (equal); writing – review and editing (equal). **Lisa Aniek de Jong:** Conceptualization (equal); formal analysis (equal); methodology (equal); supervision (equal); writing – review and editing (equal). **Didik Setiawan:** Conceptualization (equal); methodology (equal); supervision (equal); writing – review and editing (equal). **Geertruida H. de Bock:** Data curation (equal); formal analysis (equal); methodology (equal); writing – review and editing (equal). **Maarten Jacobus Postma:** Conceptualization (equal); methodology (equal); resources (equal); supervision (equal); writing – review and editing (equal).

## FUNDING INFORMATION

The research was funded by Department of Pharmacy, Universitas Islam Indonesia, and supported by a scholarship from Universitas Islam Indonesia to FDAS with reference number 628/Rek/40/DSDM/II/2020. This work also supported by the University of Groningen, University Medical Center Groningen. The funders had no role in any aspects of research or publication.

## CONFLICT OF INTEREST

The authors declare that there are no conflicts of interest.

## Supporting information


Figure S1.

Table S1.

Table S2.

## Data Availability

Data available on request due to privacy/ethical restrictions.

## References

[1] World Health Organization . Cancer incident in Indonesia. IARC. 2020;858:1‐2.

[2] Mutebi M , Anderson BO , Duggan C , et al. Breast cancer treatment: a phased approach to implementation. Cancer. 2020;126(S10):2365‐2378. 32348571 10.1002/cncr.32910

[3] Gondhowiardjo S , Soediro R , Jayalie VF , Djoerban Z , Siregar NC , Poetiray EDC . Multicenter management of breast cancer in Indonesia: ten years of experience. eJKI. 2020;8(2):121‐130.

[4] Kunkler IH , Williams LJ , Jack WJL , Cameron DA , Dixon JM . Breast‐conserving surgery with or without irradiation in women aged 65 years or older with early breast cancer (PRIME II): a randomised controlled trial. Lancet Oncol. 2015;16(3):266‐273. 25637340 10.1016/S1470-2045(14)71221-5

[5] Ohri N , Rapkin BD , Guha C , Kalnicki S , Garg M . Radiation therapy noncompliance and clinical outcomes in an urban academic cancer center. Int J Radiat Oncol Biol Phys. 2016;95(2):563‐570. 27020104 10.1016/j.ijrobp.2016.01.043

[6] The Royal College of Radiologists . The Timely Delivery of Radical Radiotherapy: Guidelines for the Management of Unscheduled Treatment Interruptions. 4th ed. London: The Royal College of Radiologists; 2019:1‐39.

[7] Bese NS , Hendry J , Jeremic B . Effects of prolongation of overall treatment time due to unplanned interruptions during radiotherapy of different tumor sites and practical methods for compensation. Int J Radiat Oncol Biol Phys. 2007;68(3):654‐661. 17467926 10.1016/j.ijrobp.2007.03.010

[8] Stoker SD , Wildeman MA , Fles R , et al. A prospective study: current problems in radiotherapy for nasopharyngeal carcinoma in Yogyakarta, Indonesia. PLoS ONE. 2014;9(1):e85959. doi:10.1371/journal.pone.0085959 24465811 PMC3900459

[9] Borras JM , Font R , Solà J , et al. Impact of non‐adherence to radiotherapy on 1‐year survival in cancer patients in Catalonia, Spain. Radiother Oncol. 2020;151:200‐205. 32771615 10.1016/j.radonc.2020.08.002

[10] Fisher B , Montague E , Redmond C , et al. Findings from NSABP protocol no. B‐04‐comparison of radical mastectomy with alternative treatments for primary breast cancer. I. Radiation compliance and its relation to treatment outcome. Cancer. 1980;46(1):1‐13. 6992972 10.1002/1097-0142(19800701)46:1<1::aid-cncr2820460102>3.0.co;2-3

[11] Gold HT , Do HT , Dick AW . Correlates and effect of suboptimal radiotherapy in women with ductal carcinoma in situ or early invasive breast cancer. Cancer. 2008;113(11):3108‐3115. 18932243 10.1002/cncr.23923

[12] Srokowski TP , Fang S , Duan Z , et al. Completion of adjuvant radiation therapy among women with breast cancer. Cancer. 2008;113(1):22‐29. 18442124 10.1002/cncr.23513 PMC4006973

[13] Li B , Brown W , Ampil F , Burton G , Yu H , McDonald J . Patient compliance is critical for equivalent clinical outcomes for breast cancer treated by breast‐conservation therapy. Ann Surg. 2000;231:883‐889. 10816632 10.1097/00000658-200006000-00013 PMC1421078

[14] Bese NS , Sut PA , Ober A . The effect of treatment interruptions in the postoperative irradiation of breast cancer. Oncology. 2005;69(3):214‐223. 16127290 10.1159/000087909

[15] Humas BPJS Kesehatan . Peserta; 2020. https://www.bpjs‐kesehatan.go.id/bpjs/pages/detail/2014/11.

[16] Vandenbroucke JP , Von Elm E , Altman DG , et al. Strengthening the reporting of observational studies in epidemiology (STROBE): explanation and elaboration. Epidemiology. 2007;18(6):805‐835. doi:10.1097/EDE.0b013e3181577511 18049195

[17] Barmawi A . Panduan Praktik Klinis Kanker Payudara; 2017.

[18] Komite Nasional Penanganan Kanker (KPKN) Kementerian Kesehatan Republik Indonesia . Panduan nasional penanganan kanker: kanker payudara; 2015.

[19] Rudat V , Nour A , Hammoud M , Abou GS . Signifikant bessere patienten compliance bei hypofraktionierter im vergleich zu konventionell fraktionierter adjuvanter strahlentherapie des mammakarzinoms: ergebnisse einer unizentrischen retrospektiven studie. Strahlenther Onkol. 2017;193(5):375‐384. doi:10.1007/s00066-017-1115-z 28233048 PMC5405099

[20] PORI . Program kerja Perhimpunan Dokter Spesialis Onkologi Radiasi Indonesia 2018–2021; 2018.

[21] de la Vega JM , Ríos B , del Río JT , Guerrero R , Castillo I , Guirado D . Management of interruptions to fractionated radiotherapy treatments: four and a half years of experience. Phys Med. 2016;32(12):1551‐1558. doi:10.1016/j.ejmp.2016.11.108 27890566

[22] Byers TE , Wolf HJ , Bauer KR , et al. The impact of socioeconomic status on survival after cancer in the United States: findings from the national program of cancer registries patterns of care study. Cancer. 2008;113(3):582‐591. doi:10.1002/cncr.23567 18613122

[23] Manajemen Pembiayaan Kesehatan . BPJS Kesehatan luncurkan program donasi, bantu warga miskin Non PBI; 2017.

[24] Zhou ZR , Mei X , Chen XX , et al. Systematic review and meta‐analysis comparing hypofractionated with conventional fraction radiotherapy in treatment of early breast cancer. Surg Oncol. 2015;24(3):200‐211. 26116397 10.1016/j.suronc.2015.06.005

