# Supplementary figures and images for: Facing problems in radiotherapy for breast cancer patients in Yogyakarta, Indonesia: A cohort retrospective study
Source: Cancer Med. 2023 Jan 20;12(7):8851–9. doi: 10.1002/cam4.5634 (PMC10134354; doi:10.1002/cam4.5634)

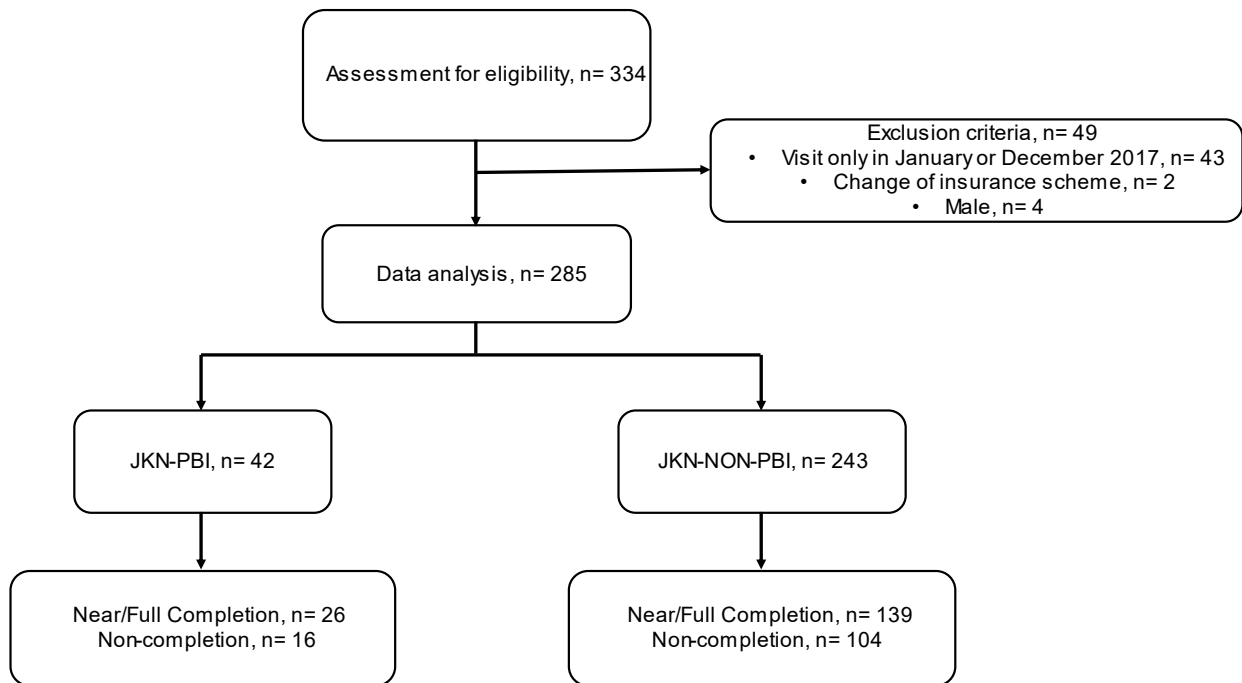

**Figure 1.** Flow diagram

Supplement: Supplementary file 1 — Figure S1. [file CAM4-12-8851-s001.pdf]
