# Supplementary material for: Facing problems in radiotherapy for breast cancer patients in Yogyakarta, Indonesia: A cohort retrospective study
Source: Cancer Med. 2023 Jan 20;12(7):8851–9. doi: 10.1002/cam4.5634 (PMC10134354; doi:10.1002/cam4.5634)
Supplement: Supplementary file 2 — Table S1. [file CAM4-12-8851-s002.docx]

Supporting Information

Table 1. Patient characteristics by completion rate

| ***Characteristic*** | **Overall (n=285)** | **Near/Full Completion**  **(n=165)** | **Incompletion**  **(n=120)** | ***p*-value** |
| --- | --- | --- | --- | --- |
| ***Gender, n (%)*** |  |  |  |  |
| *Female* | 285 (100) | 165 (100) | 120 (100) | - |
| ***Age (years), n (%)*** |  |  |  |  |
| *≤65* | 261 (91.6) | 150 (90.9) | 111 (92.5) | 0.633 |
| *>65* | 24 (8.4) | 15 (9.1) | 9 (7.5) |  |
| *Mean (SD)* | 53 (10) | 53 (10) | 52 (9) | - |
| ***Insurance scheme, n (%)*** |  |  |  |  |
| *NON-PBI JK* | 243 (85.3) | 139 (84.2) | 104 (86.7) | 0.569 |
| *PBI-JK* | 42 (14.7) | 26 (15.8) | 16 (13.3) |  |
| ***Starting day of radiotherapy, n***  ***(%)*** |  |  |  |  |
| *Monday* | 89 (31.2) | 50 (30.3) | 39 (32.5) | 0.493 |
| *Tuesday* | 79 (27.7) | 46 (27.9) | 33 (27.5) |  |
| *Wednesday* | 73 (25.6) | 40 (24.2) | 33 (27.5) |  |
| *Thursday* | 35 (12.3) | 22 (13.3) | 13 (10.8) |  |
| *Friday* | 9 (3.2) | 7 (4.2) | 2 (1.7) |  |

*Abbreviation*: SD = Standard Deviation
