# Supplementary material for: Facing problems in radiotherapy for breast cancer patients in Yogyakarta, Indonesia: A cohort retrospective study
Source: Cancer Med. 2023 Jan 20;12(7):8851–9. doi: 10.1002/cam4.5634 (PMC10134354; doi:10.1002/cam4.5634)
Supplement: Supplementary file 3 — Table S2. [file CAM4-12-8851-s003.docx]

Supporting information

Table 2. Reasons for interrupted days

| Reasons for interrupted days | Number of interrupted days | Percentage of total interrupted days | Number of patients | Percentage of total number of patients |
| --- | --- | --- | --- | --- |
| Unknown | 2,087 | 69.0 | 227 | 100.0 |
| Public holidays | 413 | 13.7 | 227 | 100.0 |
| Machine breakdown | 522 | 17.3 | 71 | 31.3 |
| Total (Median; IQR) | 3,022 (10; 6–17) | 100.0 | 227 | 100.0 |

*Abbreviation*: IQR= Interquartile range
